# ES-Cell Derived Hematopoietic Cells Induce Transplantation Tolerance

**DOI:** 10.1371/journal.pone.0003212

**Published:** 2008-09-15

**Authors:** Sabrina Bonde, Kun-Ming Chan, Nicholas Zavazava

**Affiliations:** 1 Department of Internal Medicine, University of Iowa Roy J. and Lucille A. Carver College of Medicine, Iowa City, Iowa, United States of America; 2 Department of General Surgery, Chang Gung Memorial Hospital, Chang Gung University College of Medicine, Taoyuan, Taiwan; New York University School of Medicine, United States of America

## Abstract

**Background:**

Bone marrow cells induce stable mixed chimerism under appropriate conditioning of the host, mediating the induction of transplantation tolerance. However, their strong immunogenicity precludes routine use in clinical transplantation due to the need for harsh preconditioning and the requirement for toxic immunosuppression to prevent rejection and graft-versus-host disease. Alternatively, embryonic stem (ES) cells have emerged as a potential source of less immunogenic hematopoietic progenitor cells (HPCs). Up till now, however, it has been difficult to generate stable hematopoietic cells from ES cells.

**Methodology/Principal Findings:**

Here, we derived CD45^+^ HPCs from HOXB4-transduced ES cells and showed that they poorly express MHC antigens. This property allowed their long-term engraftment in sublethally irradiated recipients across MHC barriers without the need for immunosuppressive agents. Although donor cells declined in peripheral blood over 2 months, low level chimerism was maintained in the bone marrow of these mice over 100 days. More importantly, chimeric animals were protected from rejection of donor-type cardiac allografts.

**Conclusions:**

Our data show, for the first time, the efficacy of ES-derived CD45^+^ HPCs to engraft in allogenic recipients without the use of immunosuppressive agents, there by protecting cardiac allografts from rejection.

## Introduction

Five decades ago Medawar and colleagues published an article in *Nature* [1] demonstrating that transplantation tolerance can be acquired; in these studies tolerance was strongly associated with donor leukocyte chimerism [1], [2]. Since then, it has been appreciated that engrafting allogeneic bone marrow into irradiated hosts can give the host lifelong donor-specific chimerism [3]. These hosts have hematolymphoid systems that are derived wholly or in part from donor stem cells. Such hosts are usually permanently tolerant of donor organ or tissue transplants. Thus, one can transplant fully allogeneic hematopoietic stem cells and co-transplant, for example, hearts from the hematopoietic stem cell donor to induce lifelong donor-specific acceptance of the transplant, without deleterious immunosuppression[4].

However, to achieve chimerism with allogeneic hematopoietic cells, the recipient needs extreme pre-conditioning regimens that can severely immunocompromise the patient.To avoid the severe side effects of host pre-conditioning and the dangers related to graft-versus-host disease associated with bone marrow or hematopoietic stem-cell transplantation, new data on embryonic stem (ES) cells have sparked hope that mixed chimerism and tolerance with minimal host-pretreatment can be achieved [5]. In addition to the high degree of pluripotency, ES cells can generate newly-differentiated cells that appear to have low immunogenicity, an ideal property for allogenic transplantation. Using ES cell-derived hematopoietic cells to achieve mixed chimerism would provide the opportunity to accomplish immunological tolerance without the need for harsh host pre-conditioning.

The major hindrance to exploiting ES cells has been the lack of established protocols for efficient derivation of hematopoietic cells. For example, earlier experiments by others revealed poor engraftment of ES-derived hematopoietic cells in Rag-deficient mice. Although , remarkable progress has been made in the *in vitro* development of T cells from ES cells [6], [7], this has not been the case for other hematopoietic cell lineages. Further, it has remained a challenge to obtain enough hematopoietic cells for studies on *in vitro* long-term engraftment. Recent data have now revealed that ES cells fail to engraft permanently due to their inability to self-renew [8]. However, after transfecting ES cells with the hematopoietic transcription factor HOXB4, cells can expand stably *in vitro* and *in vivo* [9]–[11].

Interestingly, the impact of HOXB4 on human ES cells appears to still be controversial. While Wang et al. [12] recently showed no expansion advantage of hematopoietic cells derived from human ES cells, Bowles and colleagues [13] saw increased expansion. Alternatively, a combination of HOXB4 and Cdx4 led to multilineage expansion of ES cell-derived hematopoietic cells in mice [14]. However, this combination has yet to be tested in human ES cells.

More recently, recombinant TAT-HOXB4 was used to expand hematopoietic stem cells up to 20-fold without the need for viral gene transfection, avoiding the potential hazards of inducing lymphoproliferative disease [15]. This property of HOXB4 can be used to expand embryonic and adult stem cells *in vitro* and *in vivo*.

Here, we exploit HOXB4, a hematopoietic transcription factor, to facilitate the derivation of large numbers of CD45^+^ cells, which were sorted and characterized. These cells poorly express MHC class I, CD80 and CD86, but no class II, allowing their transplantation across major histocompatibility barriers. Long-term engraftment was achieved, protecting allografts from immunological rejection.

Although a number of instances of successful long-term engraftment of ES-derived HPCs have been published, no data are available yet on engraftment of these cells in immunocompetent mice. For example, we have no information about how these newly-generated HPCs will compete with autologous bone marrow cells for hematopoietic niches in the recipient mice. More importantly, we also do not know whether these cells can engraft across MHC barriers and reconstitute bone marrow. The ability to do so, without severe immunosuppressive regimens, would allow studies on tolerance induction. This study focuses on the engraftment of HPCs in immunocompetent mice and proposes a strategy that could exploit available human ES cell lines to improve organ transplantation.

## Results

### HOXB4-transduced ES cells differentiate into HPCs

The derivation of multilineage hematopoietic cells from ES cells has remained difficult, limiting their use in large-scale functional studies. We previously showed that although we could successfully obtain hematopoietic cells *in vivo* and *in vitro*, the cells had only a short lifespan of up to 3–4 weeks, suggesting that they lacked self-renewal characteristics [16].

**Figure 1:**
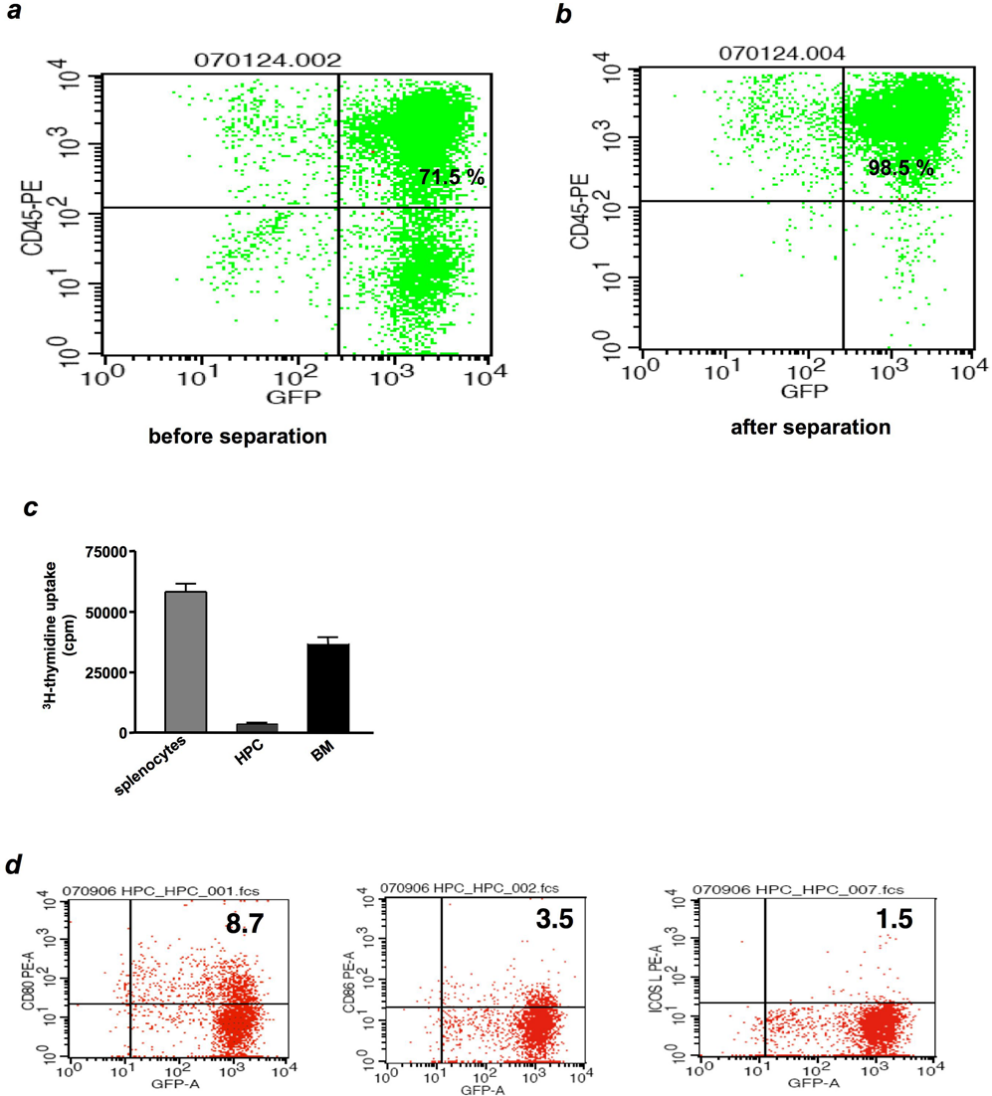
HOXB4-transduced ES cells robustly differentiate into CD45^+^. HOXB4-transduced ES cells were allowed to form embryoid bodies. The cell clusters were dismantled and subsequently treated with a hematopoietic differentiation medium. The GFP expressing cells were >70% CD45 by day 26 (A). These cells were sorted by immunomagnetic bead separation to >98% purity (B). To determine whether HPCs stimulate allogenic T cells, MRL splenocytes were co-cultured with irradiated 129SvJ splenocytes, HPCs, or bone marrow cells. Cell proliferation was determined by ^3^H-thymidine uptake. Splenocytes and bone marrow cells, but not HPCs, robustly stimulated allogenic T cells to proliferate (C). Lastly, immunophenotyping of the HPCs revealed poor expression of co-stimulatory molecules CD80, CD86, and ICOSL (D).

Here, ES cells transduced with HOXB4 were analyzed for the expression of hematopoietic markers after 26 days of differentiation. The GFP^+^HPCs expressed a whole range of leukocyte and stem cell markers including CD45, CD11b, CD31; relatively low levels of MHC class I; and no class II antigens, as we recently reported [17]. In culture, they showed a 100- to 1000-fold greater proliferation rate than non-transduced cells [17]. To further characterize these cells, we sorted the CD45-expressing cells by immunomagnetic bead separation, and measured cell purity by flow cytometry (Figure 1a–b). In general, 70–95% of the cell cultures had become CD45^+^ before the purification procedure. After purification, >98% of the cells were CD45^+^, allowing us to discard non-CD45-expressing cells. This procedure minimized the danger of teratoma formation by non-differentiated cells. Since our flow cytometric data had shown that the HPCs poorly expressed MHC antigens [17], we wondered whether they stimulate allogenic MRL splenocytes (H2^k^). For comparison, we used splenocytes and bone marrow cells, separately, as stimulator cells. HPCs failed to stimulate allogenic T cells (Figure 1c), in contrast to splenocytes and bone marrow cells, which robustly stimulated T cells. This was consistent with poor expression of MHC antigens and that of costimulatory molecules CD80, CD86 and ICOSL (Figure 1d).

### HPCs engraft permanently in immuno-deficient Rag2^−/−^γ_c_^−/−^ and in immunocompetent mice without the use of immunosuppressive agents

We previously utilized the Rag2^−/−^γ_c_^−/−^ mice to study whether the HPCs permanently engraft in an environment that lacks T, B or NK cells. Our results showed that we were able to reconstitute the Rag-deficient mice with ES cell-derived HPCs [17]. When bone marrow from these chimeric mice was transplanted into secondary recipients, mixed chimerism was >80% three months post-transplantation, suggesting that the HPCs had acquired self-renewal characteristics [17] and behaved similar to bone marrow cells (manuscript in preparation).

**Figure 2:**
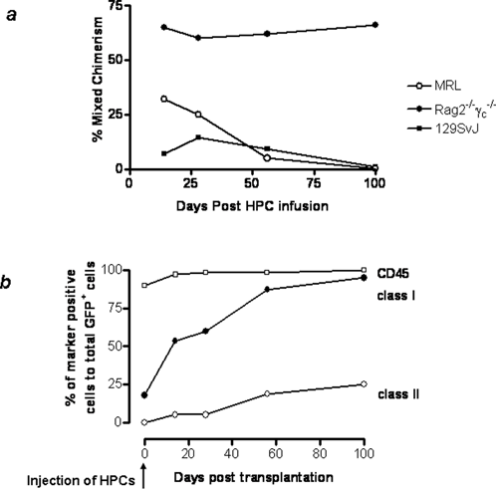
HPCs induce hematopoietic mixed chimerism. (A) 2×10^6^ HPCs were transplanted in sublethally irradiated MRL, Rag2^−/−^γ_c_^−/−^ or 129SvJ 8–12 week old mice. Mixed chimerism was monitored by measuring by flow cytometry GFP-expressing CD45^+^ cells over 100 days. (B) The expression of MHC antigens by the chimeric hematopoietic cells was determined by flow cytometry in the Rag2^−/−^γ_c_^−/−^ mice. Although the donor cells maintained a high CD45 expression from the start, there was a gradual increase of both class I and class II antigens.

Based on the low immunogenicity of the HPCs in the mixed lymphocyte cultures, we now wondered whether the HPCs would engraft in allogenic recipients. Allogenic MRL and control syngeneic 129SvJ mice were sublethally irradiated and subsequently transplanted HPCs. Mixed chimerism was measured 28 days post-transplantation (Figure 2). The long-term degree of mixed chimerism was similar between the immunocompetent MRL and 129SvJ mice, with the Rag2^−/−^γ_c_^−/−^ mice showing chimerism >70%. The lack of a difference between the syngeneic 129SvJ and allogenic MRL mice suggested that the decline in the donor cells was not due to rejection, but rather to out-competition of the HPCs by autologous bone marrow cells. However, more recent data from our lab suggest a role for NK cells (manuscript in preparation).

Despite the observed decline of HPC-derived cells in peripheral blood, 1–3% of bone marrow cells were ES-derived past 100 days, suggesting long-term persistence of donor cells. Control MRL mice were transplanted 129SvJ bone marrow cells that were rejected within 14 days. Our findings suggested immunological privilege of HPCs in the allogenic environment. This was further supported by the observation that mixed chimerism was comparable in two groups of MRL mice, one of which was treated with a single dose of an anti-CD40L antibody (data not shown). In addition, mixed lymphocyte cultures using the cells of the chimeric animals as responder cells to donor splenocytes failed to demonstrate any sensitization or clonal deletion of alloreactive T cells; i.e. T cell responses were identical to those of control animals (n = 4) (data not shown). We then tracked the expression of CD45 and class I and class II molecules on HPC-derived cells in long-term chimeric Rag2γ_c_^−/−^ mice by gating for the GFP expressing cells. Our results indeed showed a gradual increase of class I expression with time, but delayed up-regulation of class II, which appeared after day 28 (Figure 2b). We concluded that these immunological characteristics appeared to favor HPC engraftment in the allogenic setting.

### Lineage commitment of HOXB4-transduced HPCs is biased towards myeloid cells

**Figure 3:**
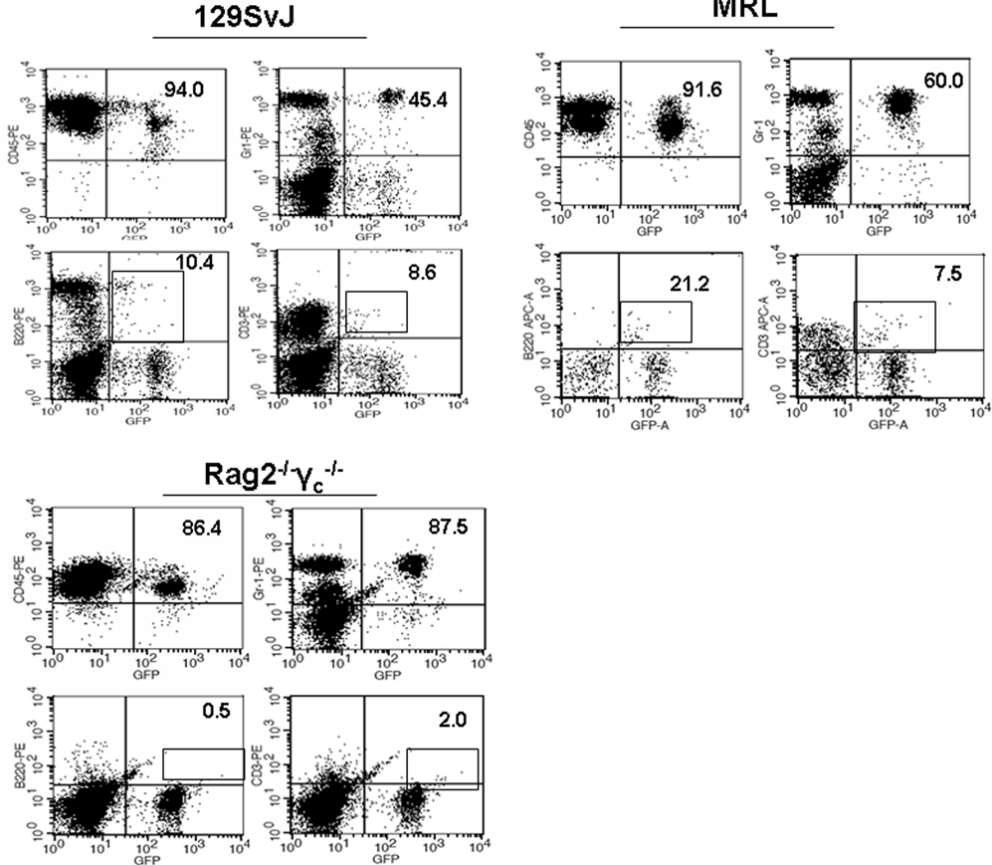
Poor T and B cell development in HPC-derived hematopoietic cells. Using flow cytometry, chimeric 129SvJ, MRL, and Rag2^−/−^γ_c_^−/−^ mice were analyzed for donor cells at 28 days post-transplantation. In all three strains, Gr-1^+^ cells were the predominant sub-population with lower cell numbers of B220- and CD3-expressing B and T cells, respectively. The percentages are calculated within the GFP^+^ population.

It has been suggested in the past that HOXB4 may interfere with lymphocytic cell development. Here, we determined the percentages of CD3^+^ and B220^+^ lymphocytes and that of Gr-1-expressing myeloid cells in either the 129SvJ, MRL or the Rag2^−/−^γ_c_^−/−^ mice 28 days post-transplantation. Interestingly, Gr-1-expressing cells were predominant in all three mouse strains, particularly in the MRL and the Rag2^−/−^γ_c_^−/−^ (Figure 3). While T and B cell development was very low in the Rag2^−/−^γ_c_^−/−^ mice, higher numbers of lymphocytes (but still lower than would be expected in bone marrow transplants) were identified in the immunocompetent mice. Of further interest was the observation that T cells in the 129SvJ and the MRLs were GFP^dim^, consistent with possible preferential GFP methylation in lymphocyte progenitors. Thus, although HOXB4 appeared to impact self-renewal and confer survival advantages to ES-derived HPCs, the development of T and B cells was impaired. However, in a previous study, we showed that these T cells successfully responded to viral antigen [17], suggesting that they were functional.

### HPCs induce donor-specific cardiac graft tolerance

**Figure 4:**
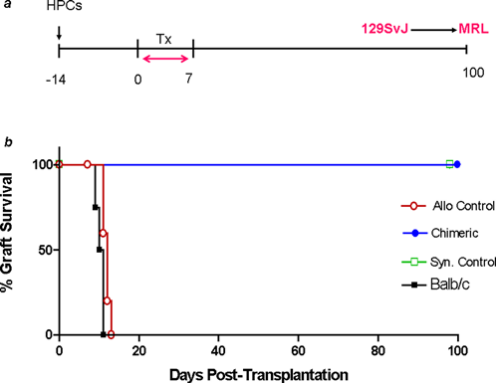
ES-derived HOXB4-expressing HPCs induce immunological tolerance to donor type cardiac allografts. A) Treatment protocol: MRL recipient mice were transplanted HPCs 14 days prior to transplantation of cardiac allografts which was performed between days 0 and 7. Cardiac function and survival were monitored by daily abdominal palpations. B) Graft survival: Chimeric MRL recipient mice tolerated donor type cardiac allografts, but not Balb/c third party allografts. Non-chimeric MRL mice acutely rejected allografts within 13 days. Syngeneic grafts were additionally used as controls.

We therefore asked whether the chimeric MRL mice were tolerant to the donor 129SvJ cardiac allografts. To do this, MRL mice were sublethally irradiated and transplanted 2×10^6^ HPCs. Mixed chimerism was first monitored after 14 days, and animals with mixed chimerism greater than 10% were transplanted donor type 129SvJ cardiac allografts. All chimeric animals accepted their allografts (n = 12) showing that established mixed chimerism was protective (Figure 4). As expected, syngeneic allografts were tolerated but Balb/c third party allografts were not. Balb/c third-party allografts were acutely rejected in chimeric animals, as were control MRL allografts in non-chimeric animals.

**Figure 5:**
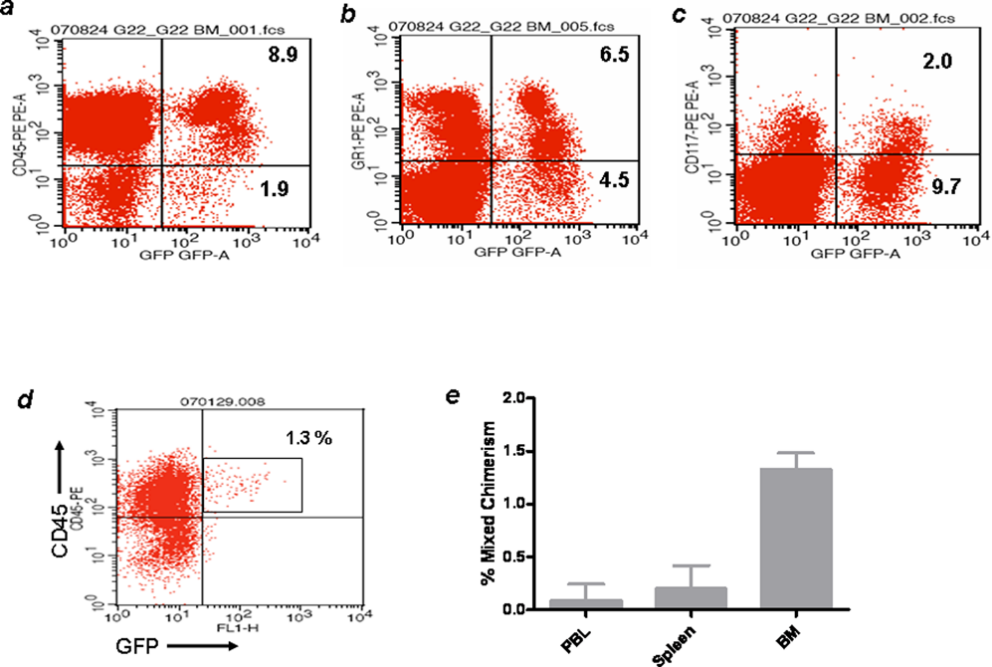
Tolerant mice maintain bone marrow mixed chimerism post-transplantation. The percentage of donor-derived hematopoietic cells in bone marrow cells of recipient tolerant mice was monitored using flow cytometry. A robust CD45^+^ cell population was detected (A) of which a large portion was Gr-1^+^ (B). Interestingly a small proportion of these cells was CD117-expressing, suggesting the presence of a truly bone marrow-resident stem cell population (C). At day 100 post-transplantation, the percentage of donor cells had decreased (D). Altogether, at day 100 post-transplantation, donor cells were hardly detectable in peripheral blood, 0.5–1% in the spleen, and 1–2% in the bone marrow (E, n = 6).

To determine a possible impact of the allograft on mixed chimerism, we measured the levels of bone marrow mixed chimerism 40 days post cardiac graft transplantation. These results, shown in Figures 5a–c, demonstrated robust mixed chimerism in the bone marrow as measured by flow cytometry. At this point, peripheral blood mixed chimerism was generally <5%. By day 100, however, mixed chimerism was undetectable in peripheral blood despite the fact that the recipient animals were tolerant to the allografts. We therefore sacrificed the mice and measured donor cells in the spleen and bone marrow. All six animals in this series showed a distinct population of CD45^+^ GFP^+^ (Figure 5d). When further stained, the cells were found to be CD117^+^Sca-1^+^. Put together, about 1.5% of bone marrow cells and <0.2% of splenic cells were GFP^+^ and therefore HPC-derived (Figure 5e). Both the spleen and peripheral blood showed much lower cell numbers than those detected in bone marrow. Thus, HPC-derived cells persisted in the bone marrow, possibly maintaining tolerance to the graft by supplying donor cells into the blood circulation. These results reinforce our notion that the decrease in mixed chimerism in the immunocompetent mice, but not in the Rag2^−/−^γ_c_^−/−^ mice, is likely due to competition for space by the recovering autologous bone marrow cells. In ongoing experiments, we are studying whether natural killer cells play a role in the engraftment of HPCs in immunocompetent mice.

### Immunohistology of tolerated allografts

**Figure 6:**
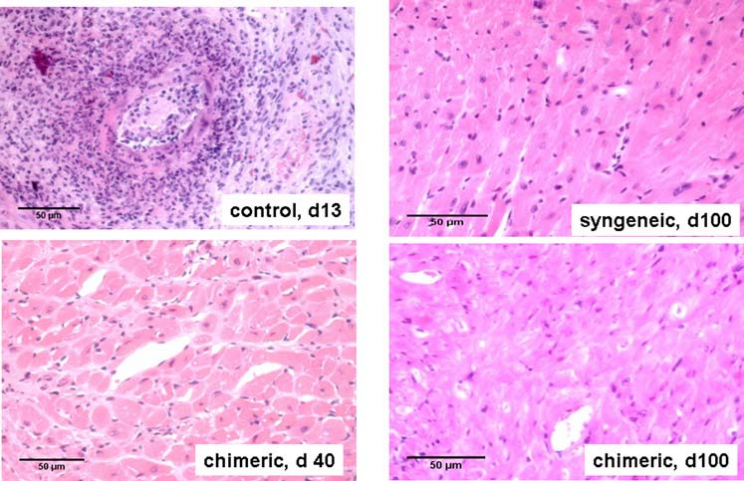
HPC-induced mixed chimerism preserves organ architecture post-transplantation. Transplanted syngeneic and allogenic animals were sacrificed at either 40 or 100 days post-transplantation and the histology of the grafts studied after H & E staining. These grafts showed preservation of the muscle architecture and lack of mononuclear cell infiltration in the syngeneic and tolerant grafts, but massive tissue destruction in the acutely rejected control grafts.

To determine the histology of the allografts, we sacrificed our transplanted animals on either day 40 or day 100 post-transplantation. As controls, syngeneic grafts at 100 days post-transplantation and acutely rejected allografts harvested 13 days post-transplantation were examined after H & E staining (Figure 6). As expected, acutely rejected allografts were massively infiltrated by mononuclear cells, whereas tolerant allografts were free of infiltrating cells at both 40 and 100 days post-transplantation. In addition, there was no evidence of intimal thickening as a sign of chronic rejection. The results were unexpected, as our animals were not treated with any immunosuppression other than the sublethal irradiation pre-HPC infusion, either pre- or post-transplantation. To rule out that this graft tolerance was not due to the irradiation alone, a control group was similarly transplanted after irradiation. All animals uniformly rejected by day 13, showing no difference to the non-irradiated controls that rejected in similar fashion.

### Induction of graft tolerance is not mediated by a deletion of alloreactive T cells, but rather is associated with intragraft FoxP3^+^CD4^+^ T cells

We wondered what mechanism was responsible for maintaining graft tolerance. One of the assays widely used in the literature is the application of splenocytes from tolerant animals as responder cells in a 4-day mixed lymphocyte culture. Balb/c splenocytes were used as third-party responder cells assuming that these would induce the same degree of T cell proliferation in tolerant and control animals. Surprisingly, there was no difference between our tolerant and control animals in the degree of T cell proliferation (data not shown). Thus, the frequency of alloreactive T cells towards the donor type alloantigen was the same in both tolerant and non-tolerant animals. This finding suggested lack of clonal deletion of alloreactive T cells.

**Figure 7:**
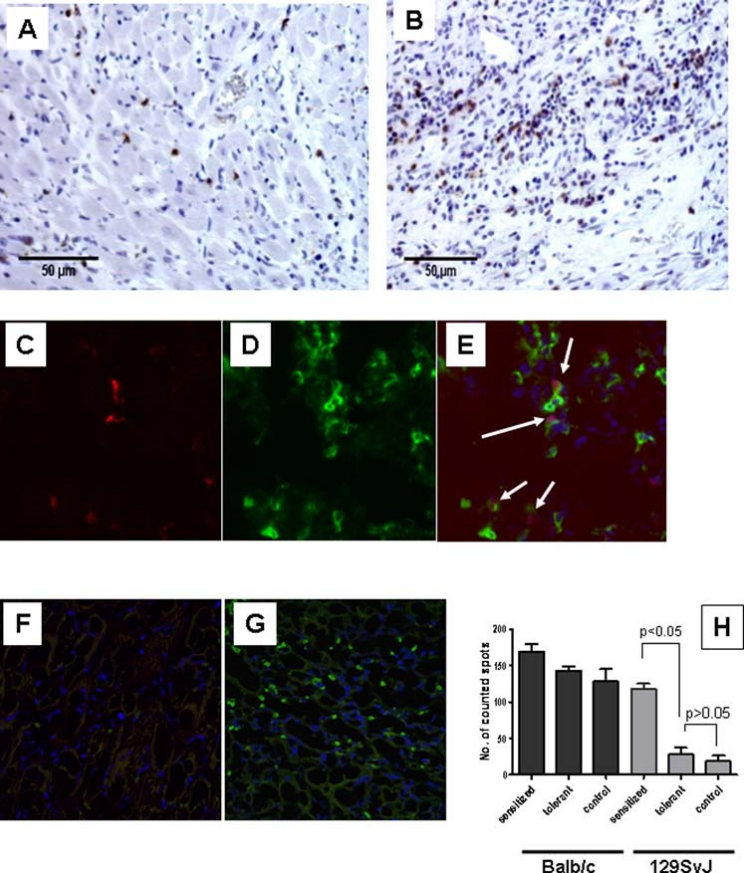
Tolerance induction is accompanied by the presence of CD4FoxP3^+^ T cells in the tolerated allografts but not in the syngeneic control grafts. A) & B) Frozen sections of tolerated cardiac allografts were stained for CD4 on day 40 post-transplantation using a peroxidase-conjugated antibody (A). A section from an acutely rejected graft was used as control (B). Positively stained cells are stained brown. C)–H) To determine whether any of the CD4^+^ T cells were Tregs, frozen sections were co-stained for CD4 (FITC) and FoxP3 (PE). In the tolerant allograft, FoxP3^+^ cells (C) and CD4^+^ (D) cells were detected, which overlapped when merged (E) suggesting that the CD4^+^ cells expressed FoxP3. In contrast, none of the syngeneic or rejected allografts showed any FoxP3-expressing cells, as reflected by the merged stains of both FoxP3 and CD4 stains in F and G, respectively. (Magnification for C–E: ×600; magnification for F and G: ×200.)H) Lastly, splenic CD4^+^ T cells from these tolerant animals were used as responder cells in an ELISPOT assay that measured IL-2 production. Controls were animals pre-sensitized with two separate IP injections of donor splenocytes and non-transplanted control animals. The sensitized animals showed a higher number of IL-2 spots against the donor splenocytes, as expected. However, the control non-sensitized and tolerant animals showed equal numbers of spots, suggesting that indeed the tolerant animals had no activated alloreactive T cells. All three groups of animals responded equally to third-party Balb/c splenocytes.

We next stained for CD4^+^ cells in the tolerated and rejected allografts. Isolated CD4^+^ cells were detected in the tolerated allografts 40 days post-transplantation (Figure 7a). In contrast, rejected allografts (13 days post-transplantation) were massively infiltrated with mononuclear cells including a much higher number of CD4-positive cells (Figure 7b). To detect regulatory T cells, we co-stained our sections by immunofluorescence for FoxP3 and CD4-expressing cells. FoxP3 expressing cells that were CD4^+^ were readily detected in tolerant allografts (Figures 7c–e). Similar stains on control syngeneic grafts and rejected allografts revealed only CD4^+^FoxP3^−^ cells (Figures 7f and 7g, respectively).

To quantitate these Tregs, we counted FoxP3^+^CD4^+^ cells in all sections. Grafts from tolerant animals had an average of 17 positive cells per 120 CD4^+^ cells on histological sections, compared to less than 1 in both the rejected or the syngeneic controls, suggesting a role for Tregs in HPC-induced tolerance. To more definitively address the role for Tregs in this model, depletion studies are ongoing.

Lastly, we isolated CD4^+^ cells from the splenocytes of the tolerant and the rejecting mice, and performed IL-2 ELISPOT analysis. ELISPOTS in tolerant animals were similar to those from control mice (p>0.05) but significantly lower than those in sensitized mice, p<0.05 (Figure 7h). These observations confirmed our results of the proliferation assays which failed to show any evidence of activated T cells in tolerant animals. However, since the ELISPOTS were nearly identical to our controls, they support lack of clonal deletion of alloreactive T cells. Responses to third-party Balb/c mice were similar in tolerant, sensitized and control non-transplanted mice showing specificity to donor-type alloantigen as would be expected.

## Discussion

ES cells provide new opportunities for developing and establishing new treatments, including transplantation tolerance induction, because of their unique characteristics: lack of MHC antigens, poor expression of co-stimulatory molecules and lack of T cells that can trigger graft-versus-host reaction. For these reasons, conditioning recipients of allogenic ES cell-derived HPCs could potentially be safer and less rigorous than conditioning recipients of bone marrow cells.

The biggest challenge, however, has been overcoming the difficulty of generating sufficient cell numbers of ES cell-derived HPCs. Earlier studies by Potocnik et al. [18], [19] showed mild engraftment of ES-derived cells in Rag-deficient mice. Such successful experiments remained rare until the description of HOXB4, a hematopoietic transcription factor that confers self-renewal properties to ES-derived cells [9]. Additionally, strategies have been described in the literature [20], [21], unfortunately, these have remained difficult to reproduce.

Here, we sought to study whether ectopically expressed HOXB4 allows the differentiation of ES cells into HPCs that can be utilized to confer transplantation tolerance. Indeed, HPC proliferation was several hundred-fold higher than in non-transduced HPCs. Importantly, these cells expressed CD45, with smaller subpopulations expressing the important hematopoietic markers CD117 and Sca-1, suggesting that these cells may have the potential to populate bone marrow and eventually replenish senescent peripheral blood hematopoietic cells. Most recently, we used HOXB4-expressing HPCs to reconstitute Rag-deficient mice, and showed robust mixed chimerism in these mice [17]. Although a number of groups have already looked at the engraftment of ES-derived HPCs in Rag2^−/−^γ_c_^−/−^ mice, there are no reports on HPC engraftment in either syngeneic or allogenic immunocompetent mice. This is important as the poor immunogenicity of these cells could be exploited for transplanting across MHC barriers with minimal conditioning of the host. Indeed, HPCs failed to stimulate allogenic T cells in proliferation assays, in contrast to splenocytes and bone marrow cells. We therefore transplanted Rag2^−/−^γ_c_^−/−^ mice, allogenic MRL mice and syngeneic 129SvJ mice with 2×10^6^ HPCs after sublethal irradiation. In Rag2^−/−^γ_c_^−/−^ mice, long-term engraftment of donor-derived HPCs was greater than 70% mixed chimerism. In contrast, engraftment in MRL mice and 129SvJ mice was generally in the range of 10–35% by day 28. While this difference was largely strain-dependent, it also reflected the fact that in the Rag2^−/−^γ_c_^−/−^ mice hematopoietic space was greater than in the immunocompetent mice. On long-term monitoring, HPC-derived hematopoietic cells remained detectable in both the allogenic and syngeneic mice, albeit declining with time. Despite the allogenic barrier, the MRL mice showed a similar pattern as the syngeneic recipients, suggesting that the engraftment was less likely to be regulated by adaptive immunological factors. The increase in expression of donor type MHC antigens on the HPC-derived hematopoietic cells was gradual. While the HPCs showed low class I expression from the beginning, gradually increasing over 60 days to >80% positive, class II antigens were first detectable at about 28 days post-transplantation.

Previously we had reported that lymphoid cell development in the Rag2^−/−^γ_c_^−/−^ mice was poor [17]. We speculated that this could be due to the poor development of the lymphoid tissues in these mice rather than the inherent inability of the HPCs to develop into lymphoid cells. Although we were able to now detect more distinct T and B cell populations in the transplanted immunocompetent MRL and 129SvJ mice, the numbers of lymphoid cells remained far less than would be expected after bone marrow transplantation. The Gr-1 expressing cells were the dominant population. This observation has been made before and appears to possibly be an influence of the overexpression of the HOXB4 gene [10], [17]. However, co-expression of cdx4 and HOXB4 in ES cells improved the generation of T and B cells [14]. This is an interesting observation as cdx4 is upstream and may therefore regulate HOXB4, reducing its negative impact on lymphopoiesis.

Once long-term mixed chimerism was established, we determined the influence of donor cells on the ability of the host's T cells to respond to alloantigen. This was first tested by using mixed lympocyte cultures. Although the T cells of chimeric animals responded to alloantigen, the response was similar to that of non-chimeric control animals, suggesting that these cells did not appear to lead to deletion of alloreactive T cells. We wondered whether this lack of stimuli could be observed in a transplantation model. Therefore, chimeric MRL mice were transplanted with donor-type 129SvJ cardiac allografts and left untreated. Third-party Balb/c cardiac allografts were transplanted in chimeric MRL mice to test for specificity. Long-term engraftment of the allogenic 129SvJ allografts in the MRL mice was observed in all chimeric recipients, but not that of third-party Balb/c allografts. These results were not expected, as recipient mice had only been sublethally irradiated and left otherwise untreated. This is the first time that *in vitro* generated HPCs were transplanted and used to establish long-term engraftment in allogeneic immunocompetent mice, resulting in the protection of donor type grafts from immunological rejection. We and colleagues previously used non-differentiated ES cell-like cells in a rat model to protect them from rejection [5]. Those experiments were, however, hampered by teratoma formation, low efficiency in engraftment, and lack of full-lineage mixed chimerism. In addition, they proved irreproducible in mice. To avoid those pitfalls here, we show that pre-differentiation and pre-sorting of HPCs avoids formation of teratomas. Further, the HPCs derived here are immunologically well-defined, allowing mechanistic studies.

Of further interest was the long-term monitoring of mixed chimerism after the cardiac transplants. By 40 days post-transplantation, peripheral mixed chimerism was less than 3% in all animals, but much higher in the bone marrow. The percentages of these cells continued to decrease and were maintained at about 1.5% in the bone marrow past day 100. These results allow us to conclude that failure to detect donor cells in peripheral blood of previously chimeric animals may not truly reflect the immunological status of these animals. Bone marrow appears to be the site where donor cells reside long-term, supplying peripheral blood with circulating donor hematopoietic cells that can maintain tolerance. We also recently showed that HPCs populated the thymus of Rag2^−/−^γ_c_^−/−^ recipient mice [17], which we believe is critical in tolerance induction. Here, we confirmed these results by detecting HPC-derived cells in the thymuses of both allogenic and syngeneic mice (data not shown). The percentage of the HPCs was, however, less than 1% but consistent, suggesting that the cells migrate into the thymus, possibly impacting the T cell repertoire of recipient mice.

In line with our contention of tolerance induction, the histology of the explanted allografts showed very low infiltration by mononuclear cells at day 40 and day 100. On histochemical staining, we noted a higher degree of CD4-expressing T cells compared to CD8^+^ cells. A higher percentage of Tregs was detected in tolerant animals than in controls, which is consistent with tolerance.

Studies on intragraft Tregs are very limited. In a non-human primate study, Haanstra et al. reported that within the graft-infiltrating lymphocytes, accepted allografts did not have greater numbers of Tregs than rejecting allografts [22]. Our results differ, which perhaps reflects the protocols used and the organ transplanted, as these investigators used a renal transplant model in a large preclinical model. These observations, coupled with the fact that we found no evidence of T cell deletion, suggest that HPCs may induce a state of non-responsiveness by inducing Tregs. We previously discussed that ES cells secrete TGF-β [16]. Thus, this property, in addition to the poor expression of MHC antigens and low expression of co-stimulatory molecules on the HPCs, might contribute to Treg development. Interestingly, when we looked at the splenocytes in these mice and compared them to controls, we were not able to detect a significant difference in the numbers of Tregs, suggesting that in the transplant model, Tregs may be within the graft-infiltrating population where they could regulate infiltrating host T cells.

Most recently, Robertson et al. reported that embryoid bodies derived from ES cells were immune privileged, requiring minimal conditioning to engraft across MHC barriers [23]. Despite the fact that embryoid bodies are a poorly-defined mixture of all three germinal layers, their data are in agreement with our findings. However, here, we used a well-defined hematopoietic cell population derived from ES cells, and we showed the kinetics of their MHC expression. The delayed and gradual upregulation of MHC antigens contributed to the immune privilege of these cells in the allogenic setting. A combination of our current protocol with a myeloablative treatment approach might further improve HPC engraftment in immunocompetent mice.

## Materials and Methods

### ES cell lines and maintenance in culture

The CCE cell line transduced with the HOXB4 gene is a generous gift from Dr. Hannes Klump (Hannover Medizinische Hochschule, Germany), and was previously described by Schiedlmeier et al. {Schiedlmeier, 2003 3370/id} and more recently by us [17]. The expression vector contained the cDNAs coding for a potential fusion protein containing the GFP (EGFP), the 2A esterase of foot-and-mouth disease virus (FMDV), and HOXB4 with an additional N-terminal hemagglutinin (HA) epitope tag (eGFP2AHOXB4). The 2A moiety mediates cotranslational separation of the growing polypeptide chain [25]. ES cells expressing EGFP were FACS-sorted and cultured. ES cells were maintained in culture according to standard conditions as we have previously described in detail [16], [26]. Briefly, ES cells were kept in standard ES cell medium containing leukemia inhibitory factor (LIF) which prevents cell differentiation. The cells were maintained on gelatinized tissue culture dishes. The culture medium was changed every 1–2 days and passaged every 2–3 days.

### Derivation and culture of HPCs

To derive HPCs, LIF was omitted from the culture media of HOXB4-transduced CCE cells to allow embryoid body formation. After five days, EBs were dissociated and seeded in serum-free medium supplemented with 100ng/ml murine stem cell factor (R & D), 10^−6^M dexamethasone (Sigma-Aldrich), 40 ng/ml IGF-1 (Promega), 2 ng/ml IL3, 5 ng/ml IL-6, and 10 ng/ml mflt-3L. In this medium, the HOXB4 gene is activated and stoichiometrically expressed at a 1∶1 ratio with GFP, so that the cells turn green as they become hematopoietic. The green fluorescence offers an excellent marker for monitoring the differentiated cells by flow cytometry post-transplantation. Cell density was kept between 2 and 4×10^6^ cells per ml.

### Mixed cell cultures

To determine whether HPCs stimulate alloreactive T cells, 5×10^5^ 129SvJ CD45^+^ HPCs, splenocytes or bone marrow cells were irradiated with 20 cGy and plated into 96-well microtiter plates in RPMI medium supplemented with 10% FCS, gentamycin and streptomycin. An equal number of MRL responder splenocytes were added and the cells cultured for 48 h before pulsing. After another 16 h, the cultures were harvested and H^3^-thymidine uptake measured in a gamma counter as we previously reported [27]. For measuring responses of chimeric or transplanted animals, splenocytes from these animals were used as responder animals similarly.

### Transplantation of HPCs and monitoring of HPC-derived hematopoietic cells

We previously established that 2×10^6^ non-differentiated ES cells per mouse are sufficient to induce mixed chimerism [16]. Here, 2×10^6^ GFP^+^CD45^+^ HPCs or bone marrow cells were infused into the supra-orbital vein of sublethally irradiated mice. Recipient animals were the allogenic MRL (H2^k^), the immunodeficient Rag2^−/−^γ_c_^−/−^ (Taconic) mice or the syngeneic 129SvJ (H2^b^) mice. The MRL and 129SvJ mice were originally purchased from Jackson Laboratory and bred at the Veterans Affairs Medical Center in Iowa City. In addition, the mice were infused with 5×10^5^ autologous bone marrow cells [10]. Mixed chimerism was monitored at 14, 28, 56, and 100 days post-transplantation. Venous blood was drawn at these time points and the cells stained with PE-conjugated antibodies against MHC class I, class II, CD45, CD3, B220 or Gr-1, which represent antibodies against myeloid and lymphoid markers. The GFP co-expression indicated HPC-derived cells. All animal experiments were approved by the Animal Care Board of the Veterans Affairs Medical Center Iowa City.

### Organ transplantation

Mice donating cardiac allografts were 8–12 week-old 129SvJ mice (H2k^b^), and the recipients were allogenic MRL mice (H2K^k^) of the same age. Cardiac transplantations were performed as previously reported {Corry, 1973 3376/id}. Cardiac function was monitored by abdominal palpations. Acutely rejected allografts ceased to beat and generally were larger, indicating cardiomyopathy.

### Post-transplant monitoring of donor cells, H & E staining and immunostaining of cardiac allografts

Acutely rejected allografts were harvested at day 13 post-transplantation. Syngeneic allografts were harvested at 100 days post-transplantation. Allogenic allografts in chimeric mice were harvested on either day 40 or day 100-post transplantation. At these time points, mixed chimerism was monitored by flow cytometry in venous blood, spleen and bone marrow. Cells were stained for CD45, B220, CD3, CD117, and Sca-1. Additionally, splenocytes of recipient animals were used as responder cells in mixed lymphocyte reactions and ELISPOT to determine responses to donor alloantigen. Paraffin sections were fixed according to standard procedure and stained with hematoxylin and eosin. To study infiltration of allografts, frozen sections were stained with peroxidase-conjugated antibodies against either CD4 or CD8 as recommended by the manufacturer (BD Biosciences, San Diego, CA). For studies on Tregs, FITC-conjugated anti-CD4 and PE-conjugated anti-FoxP3 antibodies (BD Biosciences) were used for fluorescence staining. Immunofluorescent sections were analyzed using a Bio-Rad Radience 2100 MP Confocal/Multiphoton Microscope at the Central Microscopy Research Facility at The University of Iowa.

### IL-2 ELISPOT Assay

Immunomodulation of alloresponses in chimeric animals was determined using the IL-2 ELISPOT assay as instructed by the manufacturer (BD Biosciences). Briefly, ELISPOT plates were coated with capture Ab at the prescribed concentration overnight at 4°C. Plates were subsequently washed and blocked with RPMI 1640 medium supplemented with 10% FCS for 2 h at room temperature. CD4^+^ T cells sorted by immunomagnetic beads from splenocytes of animals tolerant to donor-type cardiac allografts were plated at 10^6^ cells/well. Positive controls were CD4^+^ T cells isolated from animals prior sensitized with two immunizations of 3×10^7^ donor splenocytes over 10 days. Additional controls were splenocytes stimulated with ConA. Plates were incubated at 37°C overnight, washed, and coated with detection Ab for 2 h at room temperature. Plates were subsequently washed, coated with avidin-peroxidase for 1 h at room temperature, washed again, and developed by addition of 3-amino-9-ethylcarbazole substrate. Developed plates were dried overnight and analyzed with ImmunoSpot software. Statistical analysis was performed using the Graphpad-Prism software package, Version 4.0.

## References

[1] Billingham R, Brent L, Medawar PB (1953). “Actively acquired tolerance” of foreign cells.. Nature.

[2] Billingham R, Brent L, Medawar P (1956). The antigenic stimulus in transplantation immunity.. Nature.

[3] Weissmann A, Poulton EB, Schonland S, Shipley AE (1889). Essays upon hereditary and kindred biological problems. 25..

[4] Sykes M, Blume K, Forman S, Appelbaum FR (1999). Hematopoetic cell transplantation..

[5] Faendrich F, Lin X, Chai G, Schultze M, Ganten (2002). Preimplantation-stage stem cells induce long-term allogeneic graft acceptance without supplementary host conditioning.. Nat Med.

[6] Schmitt TM, de Pooter RF, Gronski MA, Cho SK, Ohashi PS (2004). Induction of T cell development and establishment of T cell competence from embryonic stem cells differentiated in vitro.. Nat Immunol.

[7] Schmitt TM, Zuniga-Pflucker JC (2002). Induction of T cell development from hematopoietic progenitor cells by delta-like-1 in vitro.. Immunity.

[8] Antonchuk J, Sauvageau G, Humphries RK (2002). HOXB4-induced expansion of adult hematopoietic stem cells ex vivo.. Cell.

[9] Kyba M, Perlingeiro RCR, Daley GQ (2002). HoxB4 confers definitive lymphoid-myeloid engraftment potential on embryonic stem cell and yolk sac hematopoietic progenitors.. Cell.

[10] Pilat S, Carotta S, Schiedlmeier B, Kamino K, Mairhofer A (2005). HOXB4 enforces equivalent fates of ES-cell-derived and adult hematopoietic cells.. PNAS.

[11] Zhang XB, Beard BC, Beebe K, Storer B, Humphries RK (2006). Differential effects of HOXB4 on nonhuman primate short- and long-term repopulating cells.. PLoS. Med.

[12] Wang L, Menendez P, Shojaei F, Li L, Mazurier F (2005). Generation of hematopoietic repopulating cells from human embryonic stem cells independent of ectopic HOXB4 expression. J Exp Med.

[13] Bowles KM, Vallier L, Smith JR, Alexander MRJ, Pedersen RA (2006). HOXB4 overexpression promotes hematopoietic development by human embryonic stem cells.. Stem Cells.

[14] Wang Y, Yates F, Naveiras O, Ernst P, Daley GQ (2005). Embryonic stem cell-derived hematopoietic stem cells.. PNAS.

[15] Krosl J, Austin P, Beslu N, Kroon E, Humphries (2003). In vitro expansion of hematopoietic stem cells by recombinant TAT-HOXB4 protein.. Nat Med.

[16] Bonde S, Zavazava N (2006). Immunogenicity and engraftment of mouse embryonic stem cells in allogeneic recipients.. Stem Cells.

[17] Chan K, Bonde S, Klump H, Zavazava N (2008). Hematopoiesis and immunity of HOXB4-transduced embryonic stem cells-derived hematopoietic progenitor cells.. Blood.

[18] Potocnik AJ, Kohler H, Eichmann K (1997). Hemato-lymphoid in vivo reconstitution potential of subpopulations derived from in vitro differentiated embryonic stem cells.. PNAS.

[19] Potocnik AJ, Nerz G, Kohler H, Eichmann K (1997). Reconstitution of B cell subsets in Rag deficient mice by transplantation of in vitro differentiated embryonic stem cells.. Immuno Lett.

[20] Burt RK, Verda L, Kim DA, Oyama Y, Luo K (2004). Embryonic stem cells as an alternate marrow donor source: engraftment without graft-versus-host disease.. J Exp Med.

[21] Honig GR, Li F, Lu SJ, Vida L (2004). Hematopoietic differentiation of rhesus monkey embryonic stem cells.. Blood Cells Mol Dis.

[22] Haanstra KG, Wubben JA, Korevaar SS, Kondova I, Baan CC (2007). Expression patterns of regulatory T-cell markers in accepted and rejected nonhuman primate kidney allografts.. Am J Transplant.

[23] Robertson NJ, Brook FA, Gardner RL, Cobbold SP, Waldmann H (2007). Embryonic stem cell-derived tissues are immunogenic but their inherent immune privilege promotes the induction of tolerance.. PNAS.

[24] Schiedlmeier B, Klump H, Will E, Arman-Kalcek G, Li Z (2003). High-level ectopic HOXB4 expression confers a profound in vivo competitive growth advantage on human cord blood CD34+ cells, but impairs lymphomyeloid differentiation.. Blood.

[25] Just U, Stocking C, Spooncer E, Dexter TM, Ostertag W (1991). Expression of the GM-CSF gene after retroviral transfer in hematopoietic stem cell lines induces synchronous granulocyte-macrophage differentiation.. Cell.

[26] Chan KM, Bonde S, Klump H, Zavazava N (2008). Hematopoiesis and immunity of HOXB4-transduced embryonic stem cell-derived hematopoietic progenitor cells.. Blood.

[27] Zavazava N, Krönke M (1996). Soluble HLA class I molecules induce apoptosis in alloreactive cytotoxic T lymphocytes.. Nat Med.

[28] Corry R, Winn H, Russell P (1973). Primarily vascularized allografts of hearts in mice.. Transplantation.

